# Supplementary material for: Profiling of the plasma proteome across different stages of human heart failure
Source: Nat Commun. 2019 Dec 20;10:5830. doi: 10.1038/s41467-019-13306-y (PMC6925199; doi:10.1038/s41467-019-13306-y)
Supplement: Supplementary file 1 — Supplementary Information [file 41467_2019_13306_MOESM1_ESM.pdf]

# Profiling of the plasma proteome across different stages of human heart failure

**Supplementary information**

Anna Egerstedt, John Berntsson, Maya Landenhed Smith, Olof Gidlöf, Roland Nilsson, Mark Benson, Quinn S. Wells, Selvi Celik, Carl Lejonberg, Laurie Farrell, Sumita Sinha, Dongxiao Shen, Jakob Lundgren, Göran Rådegran, Debby Ngo, Gunnar Engström, Qiong Yang, Thomas J. Wang, Robert E. Gerszten, J. Gustav Smith

Contents:

Supplementary Figure 1: Protein characteristics.

Supplementary Figure 2: P-value distribution for protein associations with heart failure.

Supplementary Figure 3: Comparison of Somascan assay with immunoassays.

Supplementary Figure 4. Distribution of protein concentrations across study populations.

Supplementary Figure 5: Spatial transcriptomic expression of cardiac proteins.

Supplementary Figure 6: Cell-type specific expression of cardiac proteins in murine hearts.

Supplementary Figure 7: QQ plots from genome-wide association studies of identified proteins.

Supplementary Figure 8: Manhattan plots from genome-wide association studies of identified protein

Supplementary Table 1: Comparison of risk factor estimates for incident HF in the full population-based cohort and the nested case-cohort sample.

Supplementary Table 2: Baseline characteristics of clinical heart failure cohorts.

Supplementary Table 3: Association with proteins from established pathways.

Supplementary Table 4: Association with manifest heart failure for proteins identified for heart failure development.

Supplementary Table 5: Correlation of proteins associated with heart failure development.

Supplementary Table 6: Gene set enrichment analysis for manifest heart failure.

Supplementary Table 7: Evidence of cardiac origin.

Supplementary Table 8: Results from genome-wide association analyses.

**Supplementary Figure 1. Protein characteristics.**

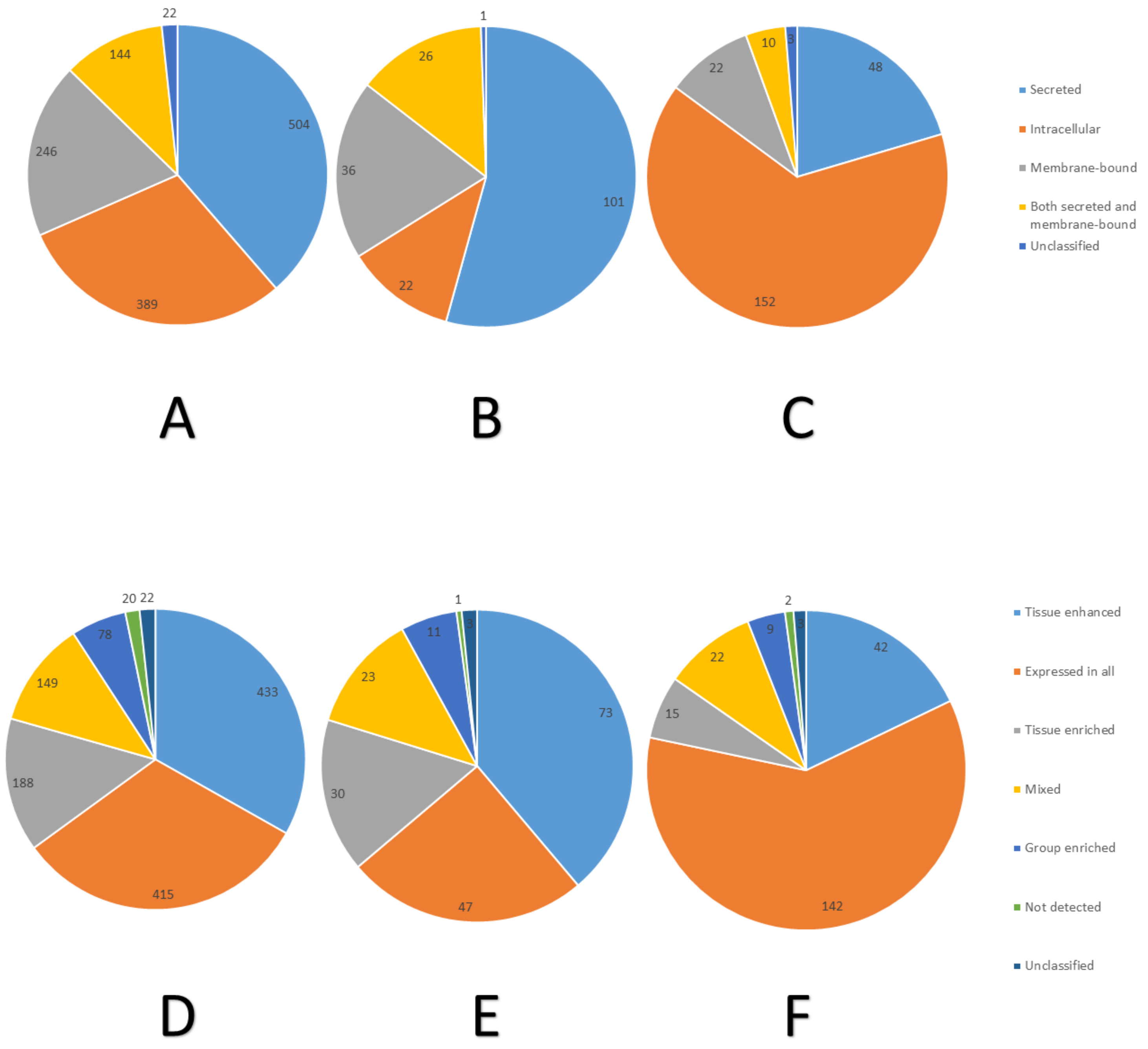


Distribution of protein classes (A-C) and tissue expression (D-F), among all 1,305 proteins assayed (A, D), the subset of 186 proteins higher in patients with HF (B,E), and among 235 proteins lower in patients with HF (C,F). Protein locations were computationally predicted based on structural features, as part of the Human Protein Atlas (HPA, https://www.proteinatlas.org/humanproteome/secretome, accessed sept 9, 2018). For membrane proteins, features indicating transmembrane regions included

length, amino acid properties and hydrophobicity based on combination of seven algorithms. For secreted proteins, three algorithms for prediction of signal peptides were combined. Intracellular proteins were defined as lacking both transmembrane regions and signal peptides. A subset of proteins included both signal peptides and transmembrane regions and a small subset was unavailable in the HPA database. Information on tissue enrichment was also obtained from the HPA, based on RNA-sequencing of 64 cell lines and 37 tissues. Tissue expression was classified by the HPA into six categories: tissue enriched (expression in one tissue at least five-fold higher than all others), group enriched (five-fold higher in 2-7 tissues compared to all other), tissue enhanced (five-fold higher in one or more tissues compared to the mean of all tissues), expressed in all (≥1 transcript per million in all tissues), not detected (<1 transcript per million in all tissues), and mixed (detected in at least one tissue and not in the other categories).

**Supplementary Figure 2. P-value distribution for protein associations with heart failure.**

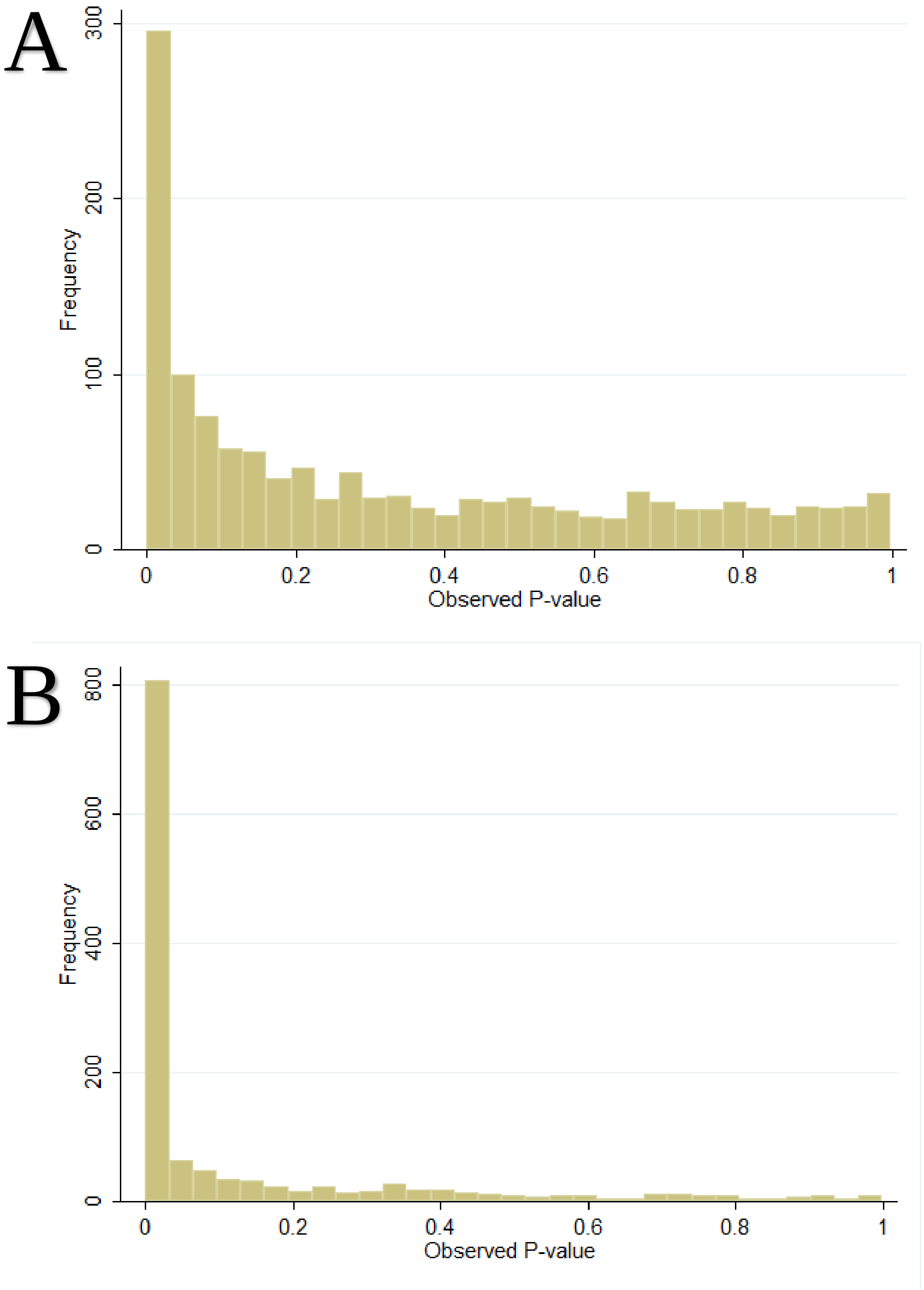


Histogram showing distribution of p-values for association tests of 1,305 proteins with incident heart failure in a population-based cohort (panel a) and manifest heart failure (panel b). A marked excess of proteins with low p-values is observed with both but most markedly with manifest heart failure, consistent with a large number of association signals.

**Supplementary Figure 3. Comparison of Somascan assay and immunoassays.**

A

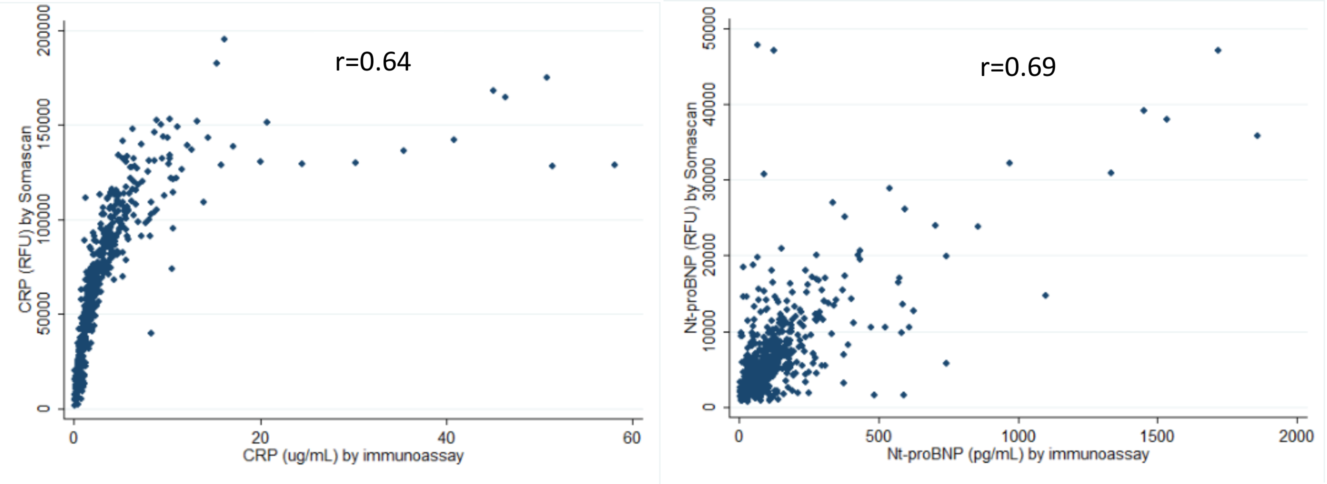


B

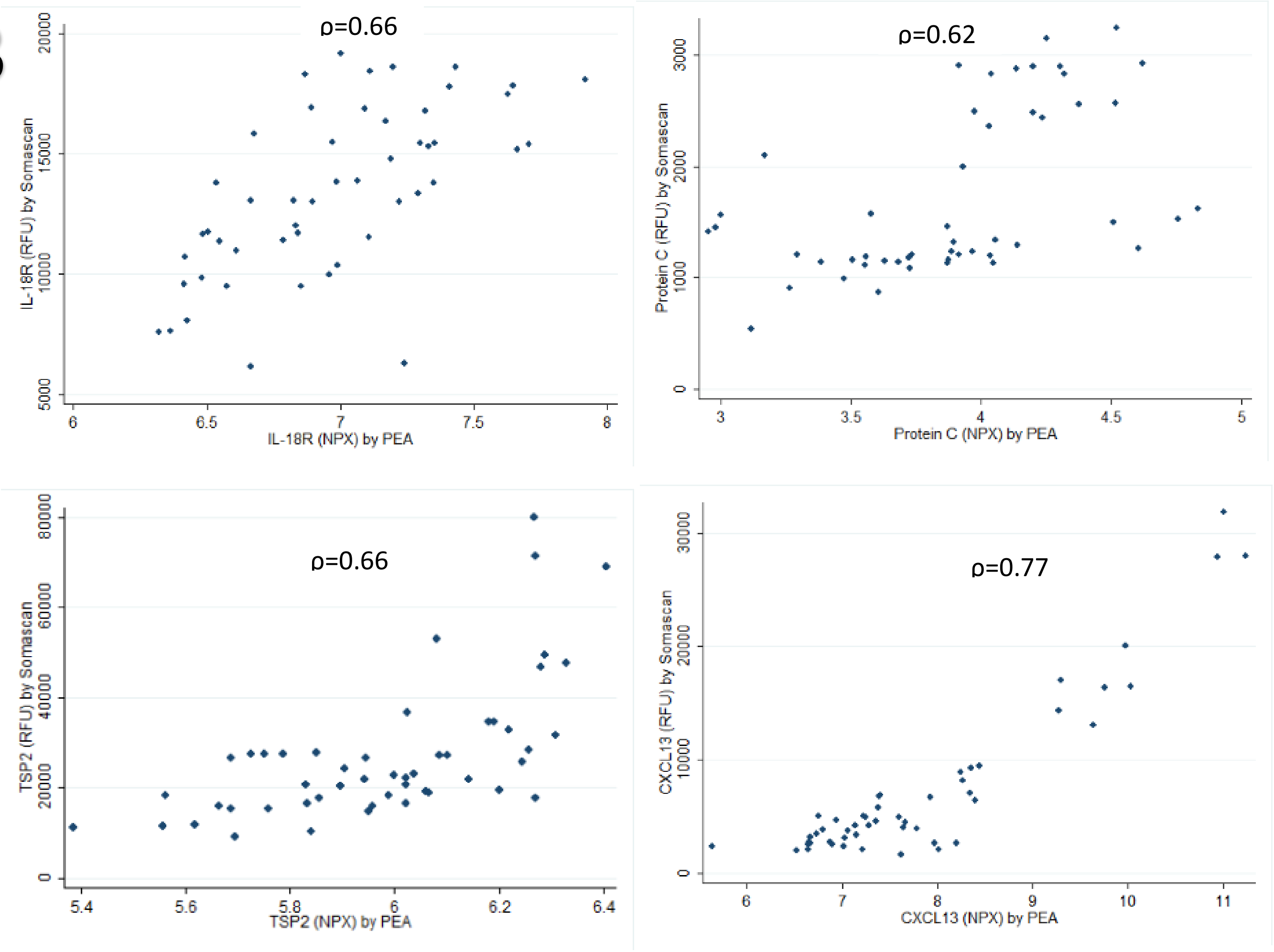

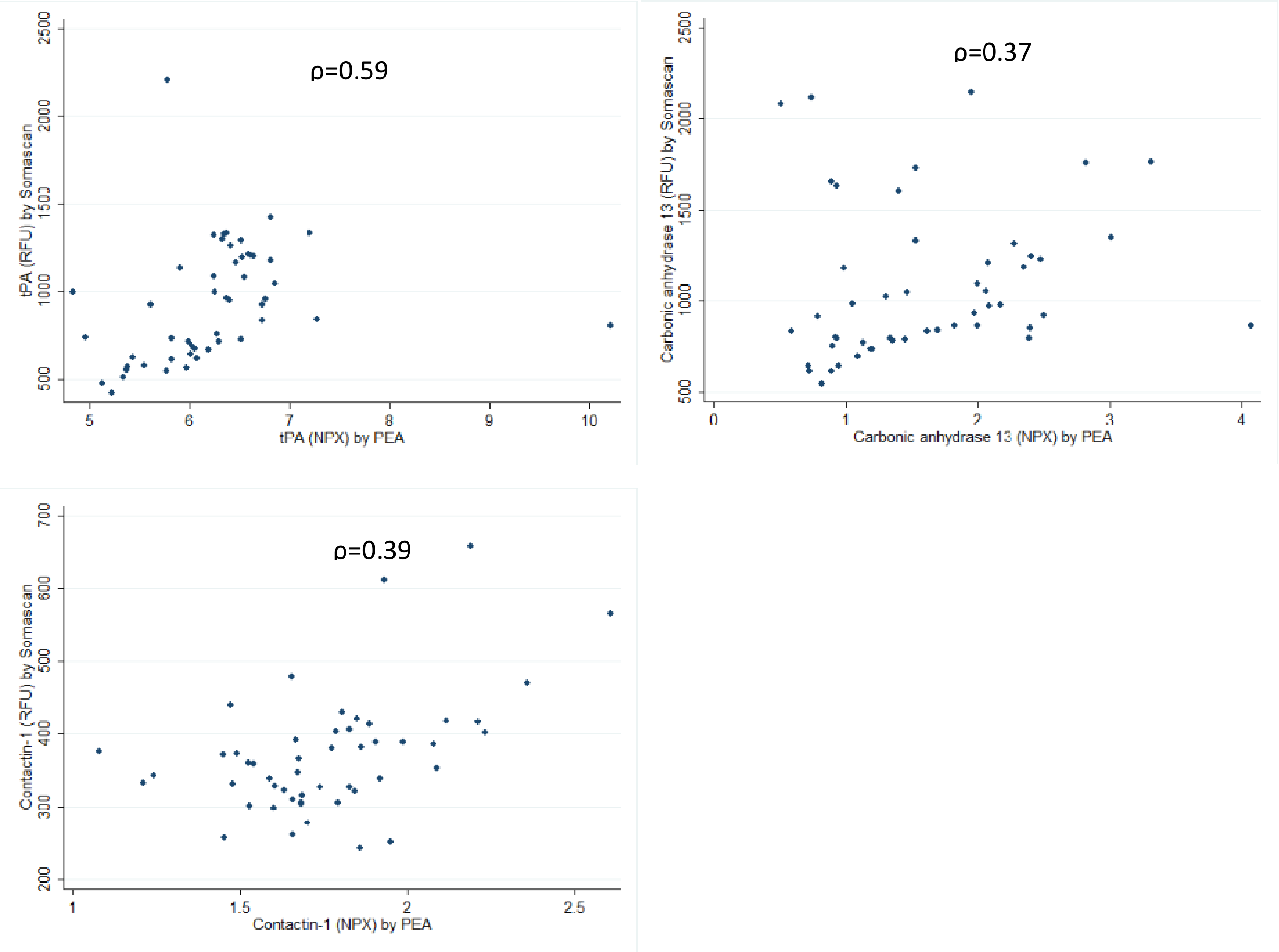


Panel A shows scatterplots for C-reactive protein (CRP) and N-terminal pro-B-type natriuretic peptide (Nt-proBNP) measured using Somascan (in relative fluorescent units, RFUs) or established immunoassays (in pg/mL) in the case-cohort sample from the MDCS. Pearson's correlation coefficients are presented (both $p<0.05$). CRP was measured by a high-sensitivity assay (Roche Diagnostics, Basel, Switzerland). Nt-proBNP was measured using the automated Dimension Vista Intelligent Lab System method (Siemens Healthcare Diagnostics Inc, Deerfield, Illinois, USA).

Panel B shows scatterplots for 7 proteins measured using Somascan or a proximity extension assay (PEA; Olink Proteomics, Uppsala, Sweden) in 50 samples: 10 samples from patients with myocardial infarction, and 4 samples (0, 10 min, 1 hour and 24 hours after) each from 10 patients with hypertrophic cardiomyopathy undergoing septal alcohol ablation. Spearman's rank correlation coefficients are presented (all $p<0.05$).

**Supplementary Figure 4. Distribution of protein concentrations across study populations.**

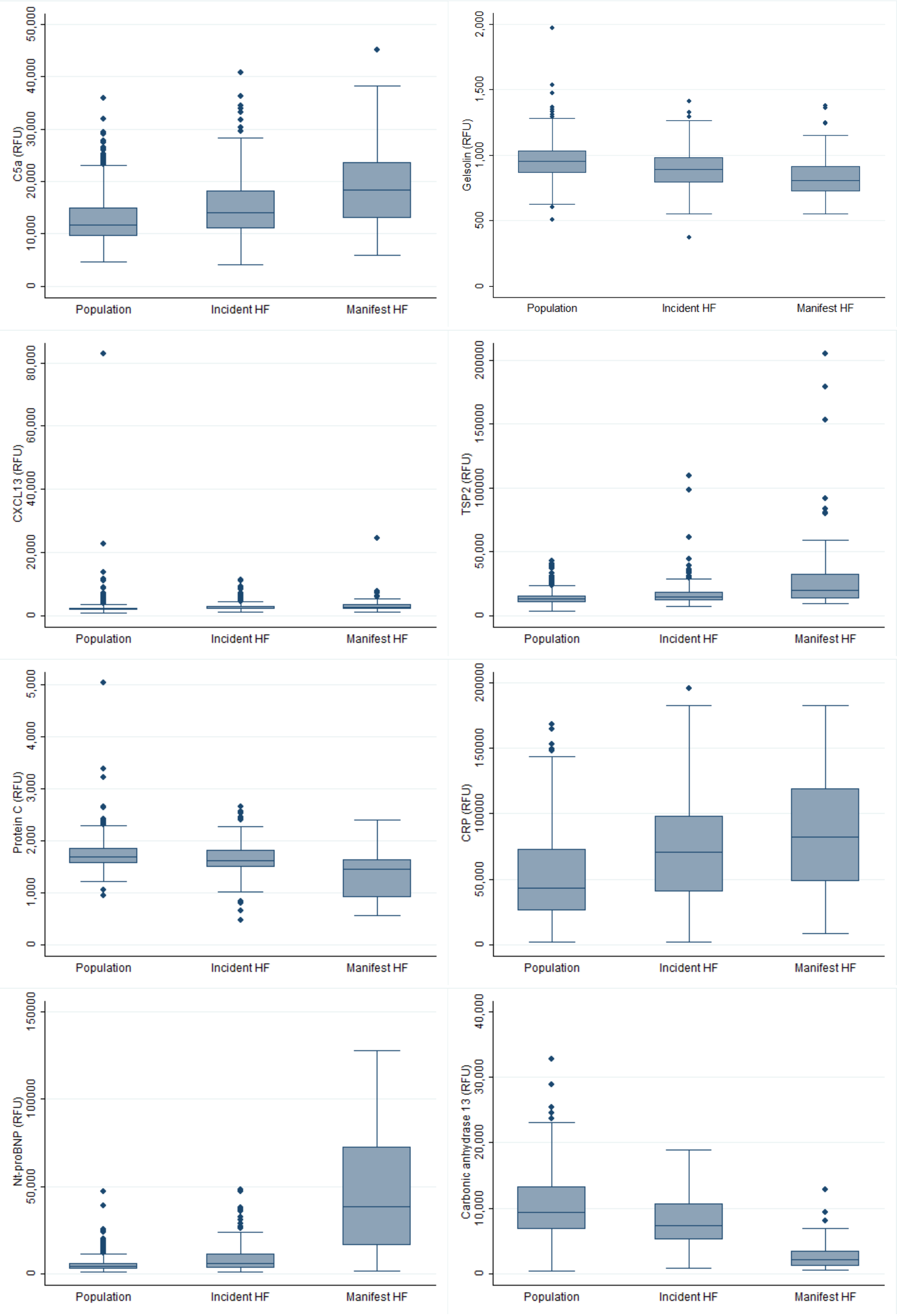

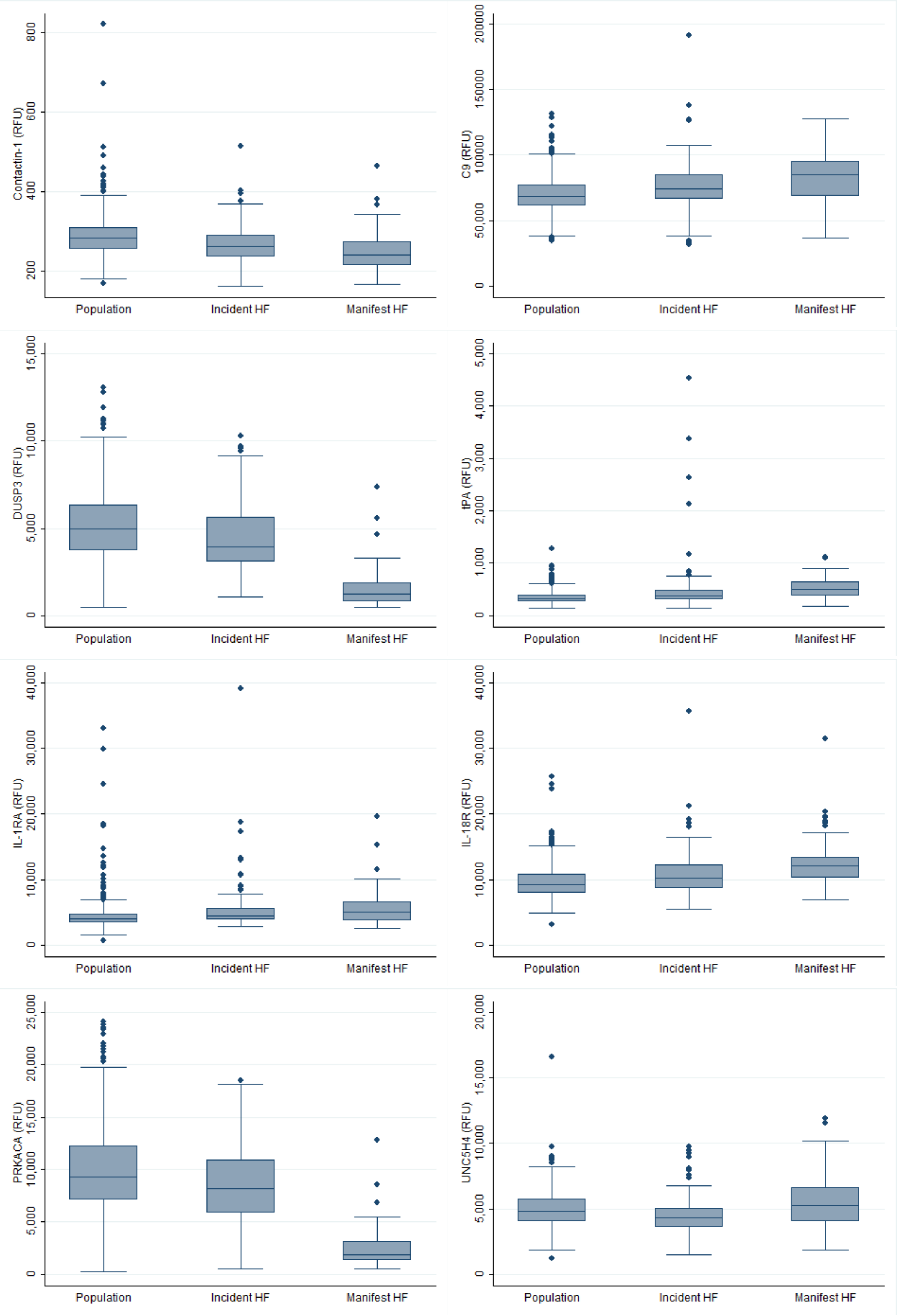


The box centre line indicates the median, box bounds represent 25th and 75th percentiles, and the whiskers are the most extreme values within 1.5 x the interquartile range from the nearer quartile.

**Supplementary Figure 5: Spatial transcriptomic expression of cardiac proteins.**

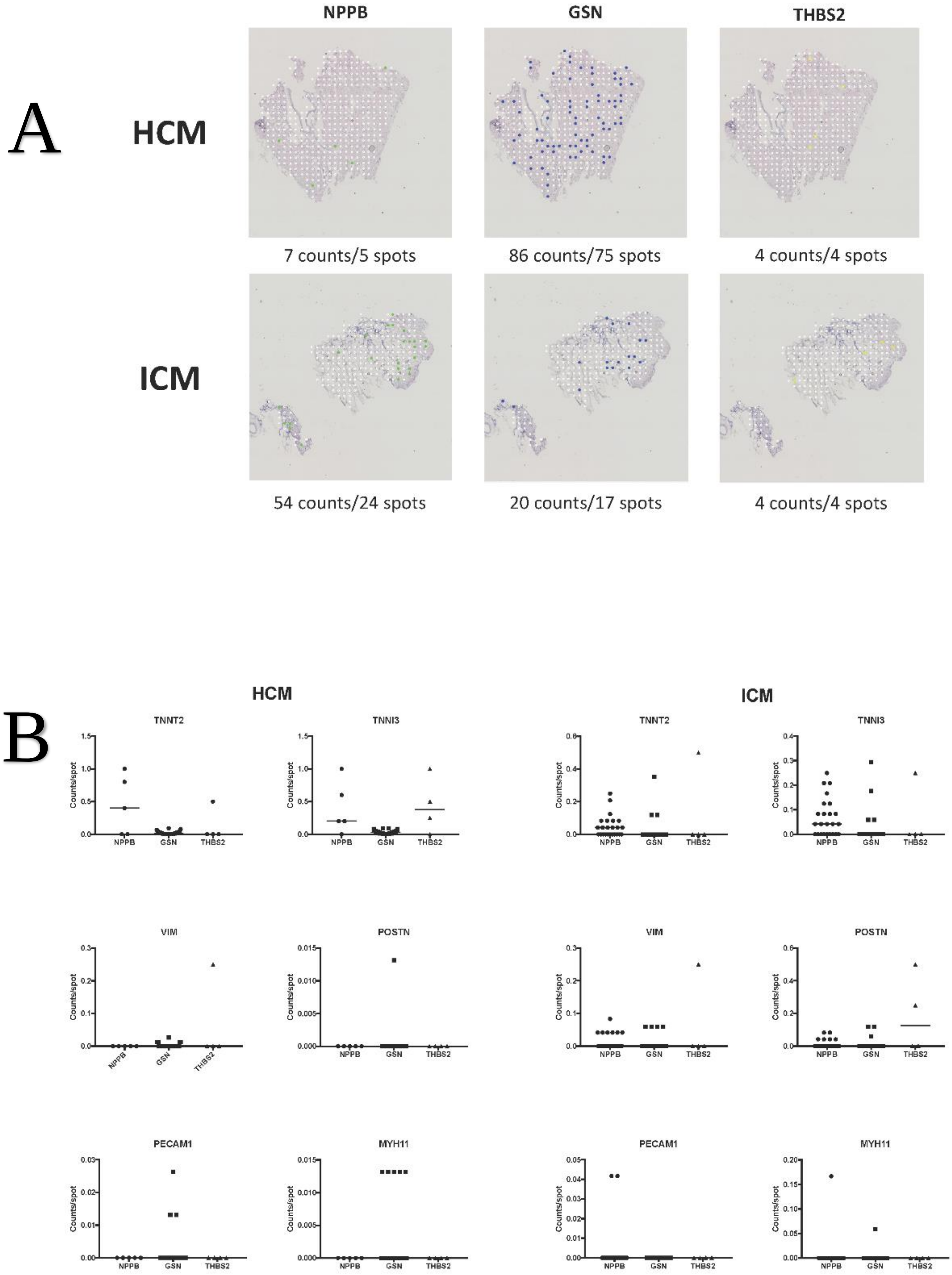


Panel (A) Myocardial expression across spatially distinct spots on tissue sections for Nt-proBNP (*NPPB*), thrombospondin-2 (*THBS2*) and gelsolin (*GSN*), for which evidence of cardiac origin was observed, in two patients with heart failure of different etiologies: ischemic (ICM) and hypertrophic

(HCM) cardiomyopathy. Panel (B) Coexpression was tested for spots expressing at least one count of each transcript with markers specific for cardiomyocytes (*TNNT2*, *TNNI3*), fibroblasts (*VIM*, *POSTN*), endothelial cells (*PECAM1*) and vascular smooth muscle cells (*MYH11*). Expression of IL18R, and presence of leukocytes (*PTPRC* [CD45]), was below the level of detection for all spots and was not included.

**Supplementary Figure 6: Cell-type specific expression of cardiac proteins in murine hearts.**

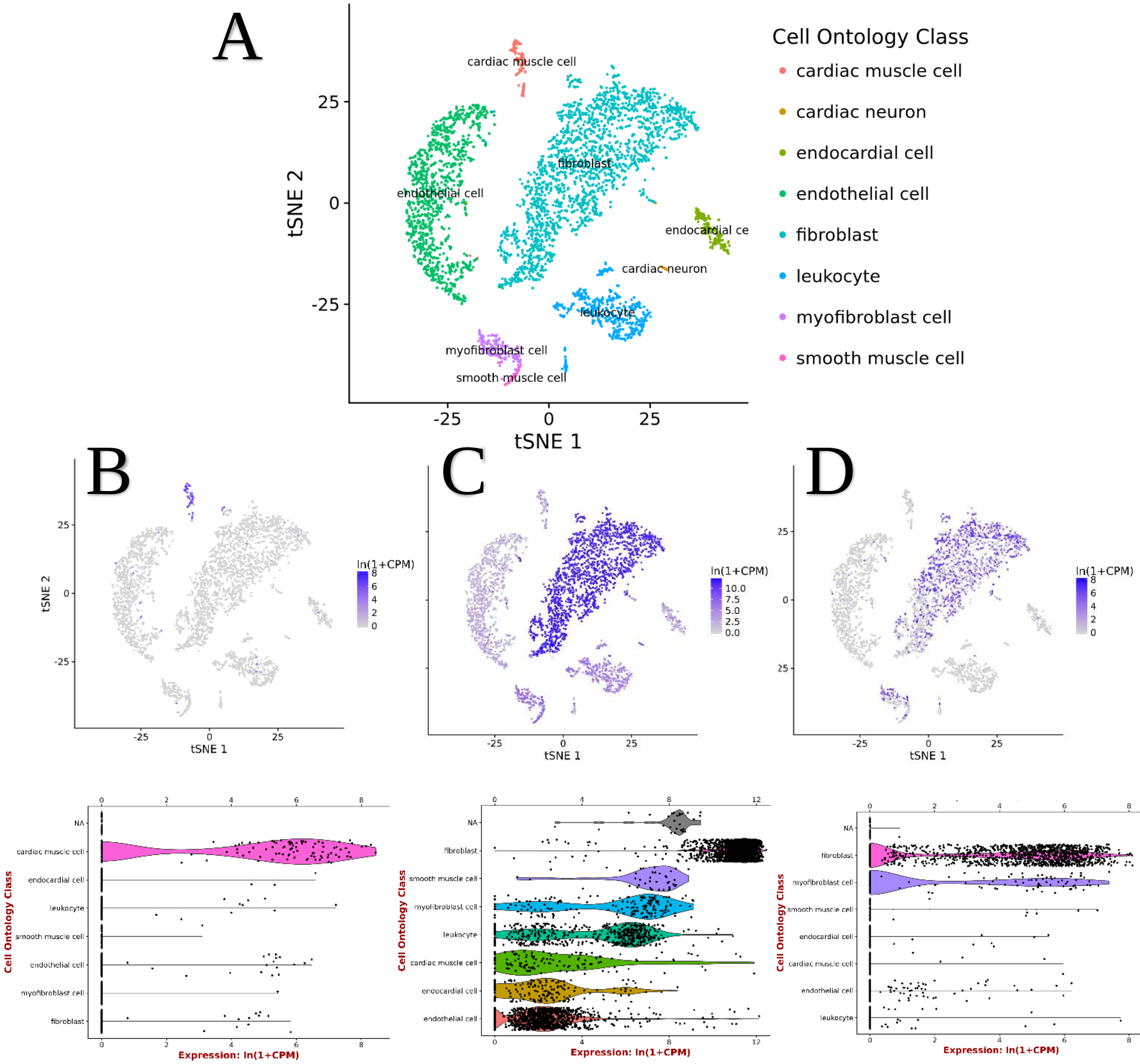


Single-cell full length transcript analysis of mouse hearts generated by SMART-Seq2 RNA-sequence libraries prepared from individually fluorescence-activated cell sorter (FACS) sorted cells within the Tabula Muris project. (https://tabula-muris.ds.czbiohub.org/, retrieved on March 18, 2019) Panel A shows cell clustering for murine hearts based on t-distributed stochastic neighbor embedding (tSNE) in two dimensions. The upper panels for each of *NPPB* (B), *GSN* (C) and *THBS2* (D) indicate

expression of each transcript in blue, while the lower panels show violin plots with distribution of gene expression across cell types, expressed in transformed counts per million (CPM).

**Supplementary Figure 7: QQ plots from genome-wide association studies of identified proteins.**

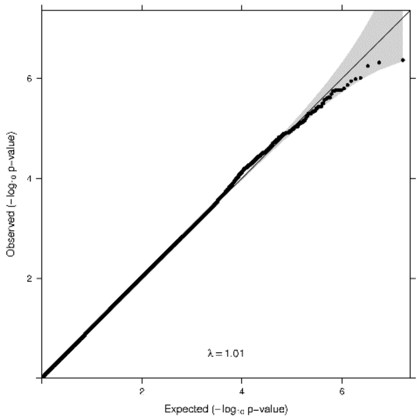


CXCL13

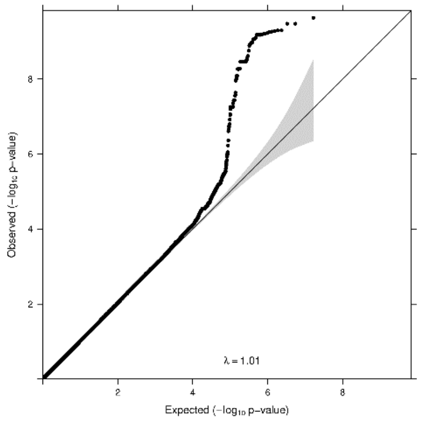


C5a

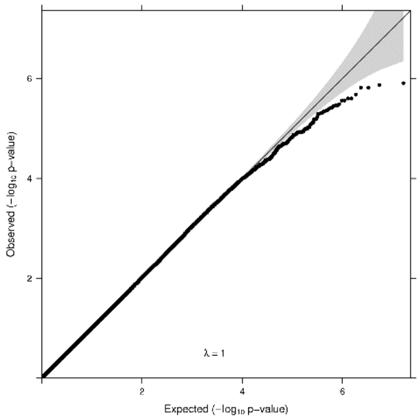


C9

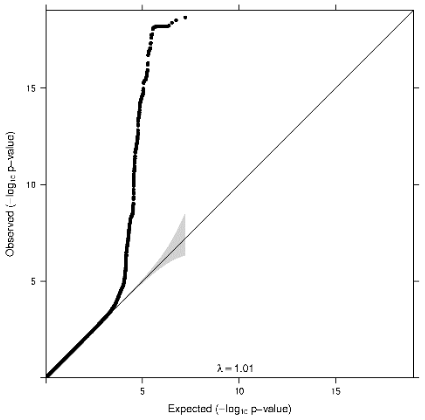


Carbonic anhydrase XIII

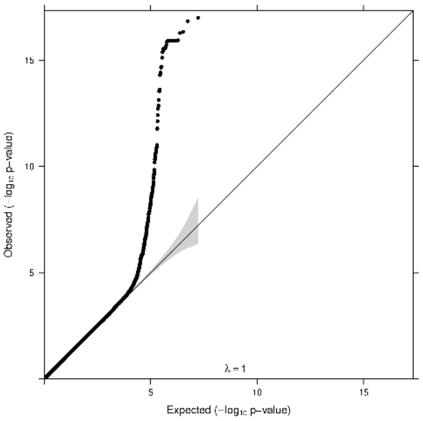


Contactin-1

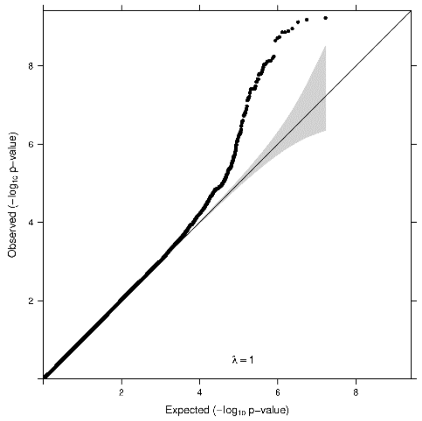


CRP

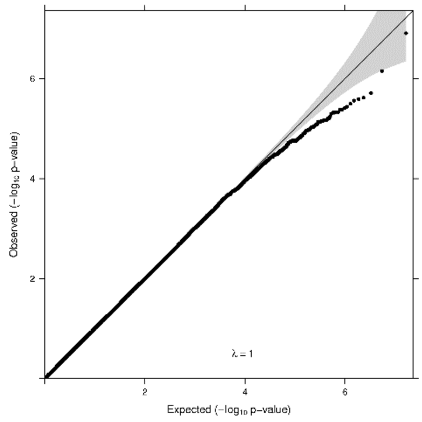


DUSP3

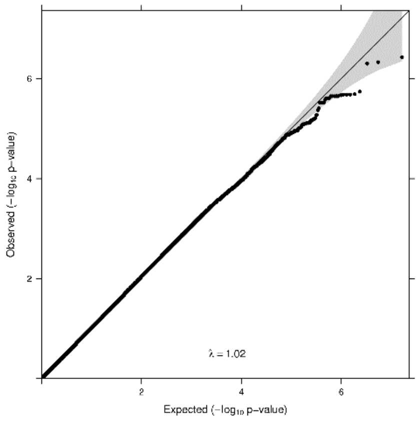


Gelsolin

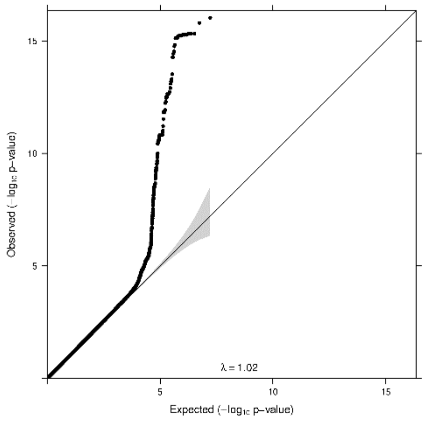


IL-1RA

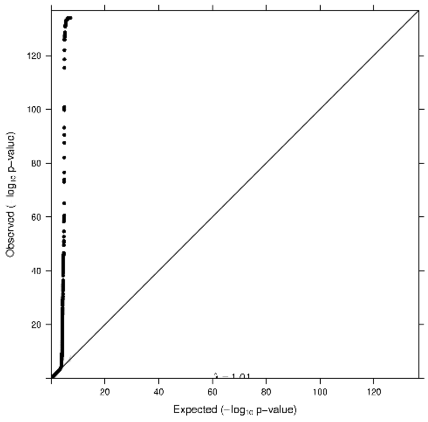


IL-18R

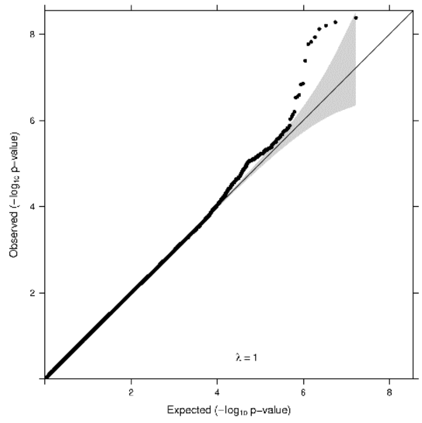


Nt-ProBNP

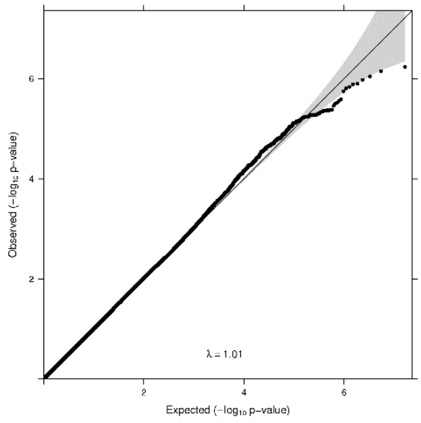


PRKACA

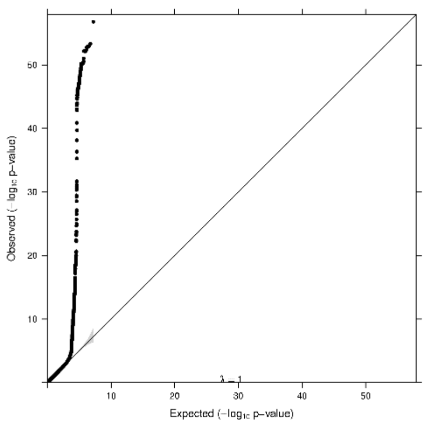


Protein C

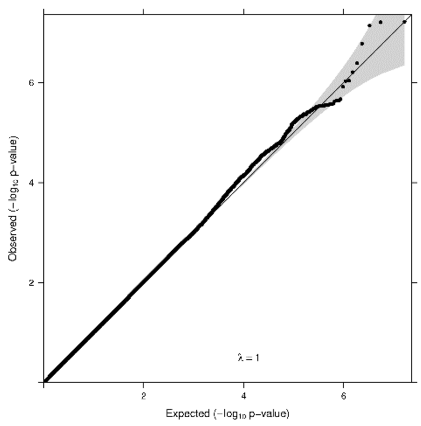


tPA

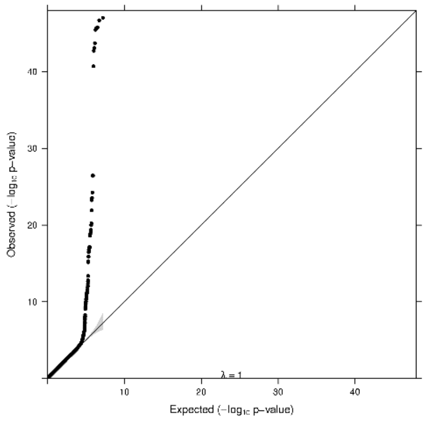


TSP2

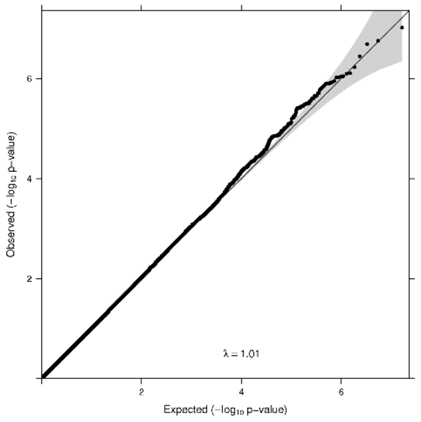


UNC5H4

**Supplementary Figure 8: Manhattan plots from genome-wide association studies of identified proteins.**

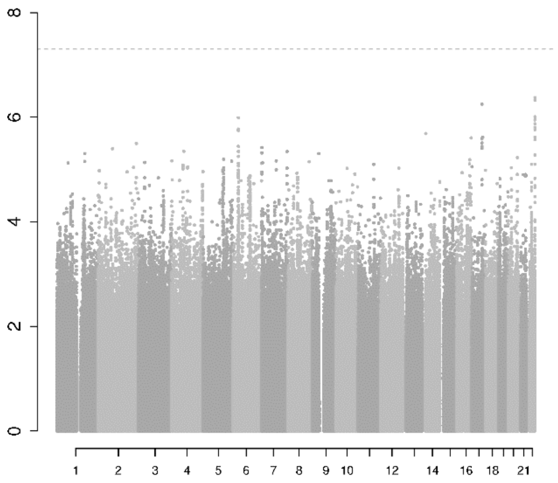


CXCL13

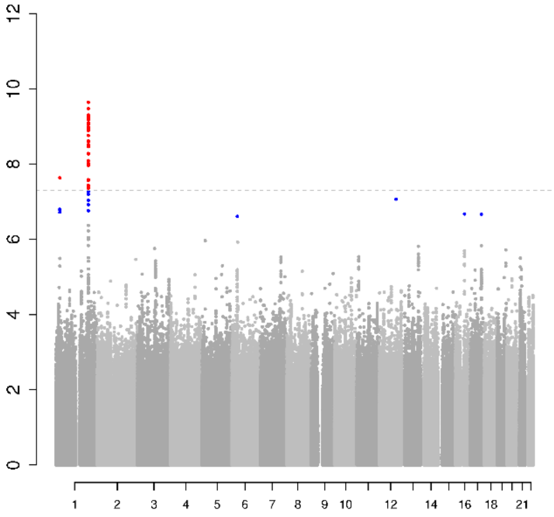


C5a

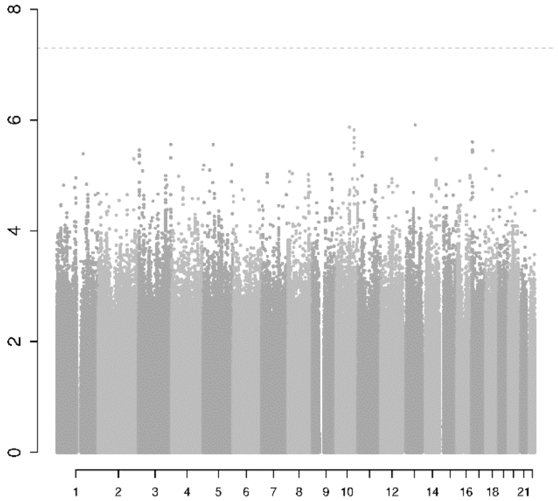


C9

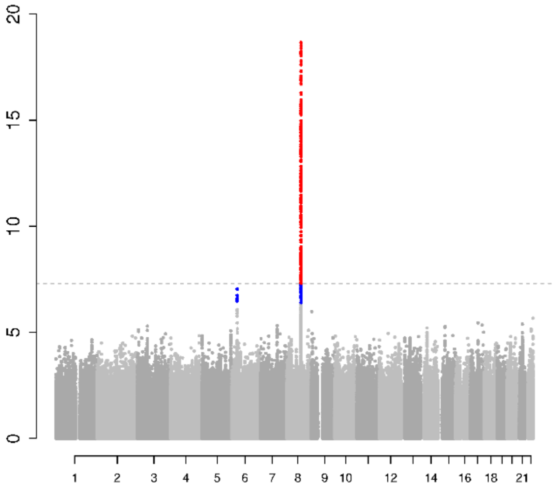


Carbonic anhydrase XIII

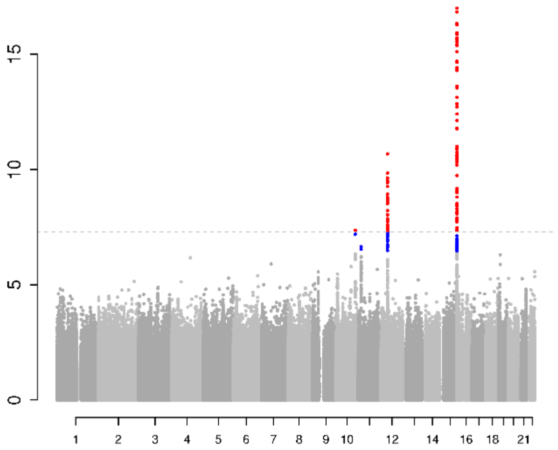


Contactin-1

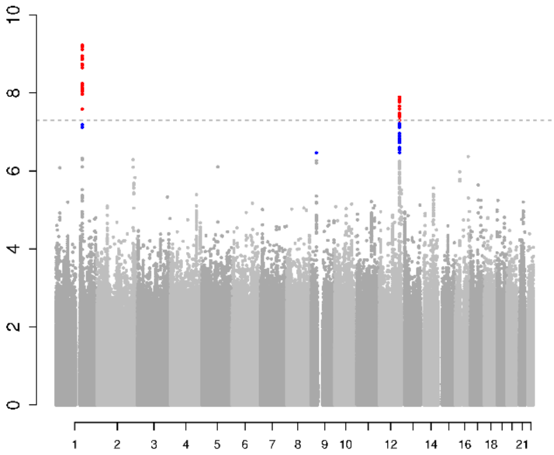


CRP

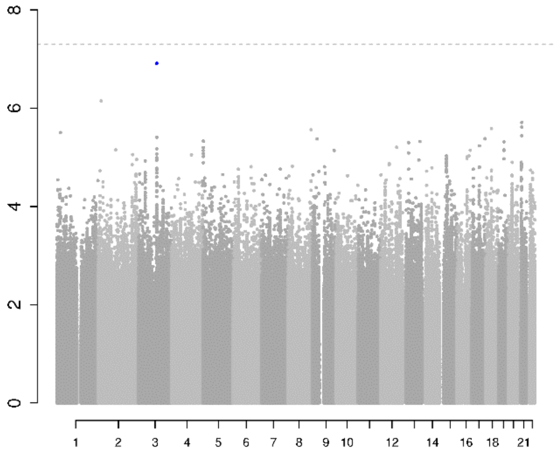


DUSP3

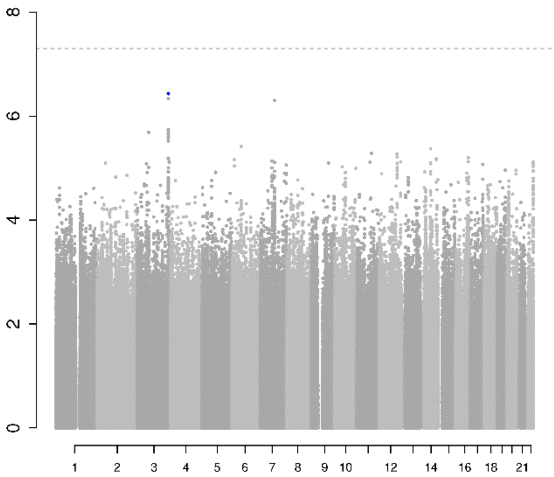


Gelsolin

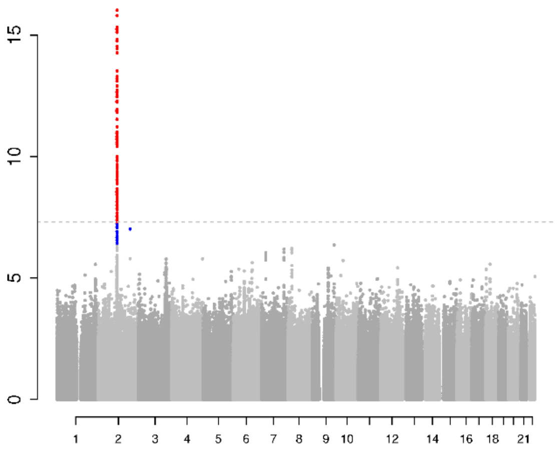


IL-1RA

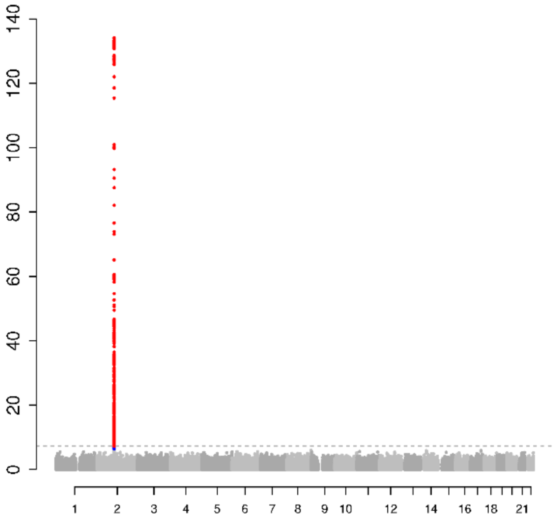


IL-18R

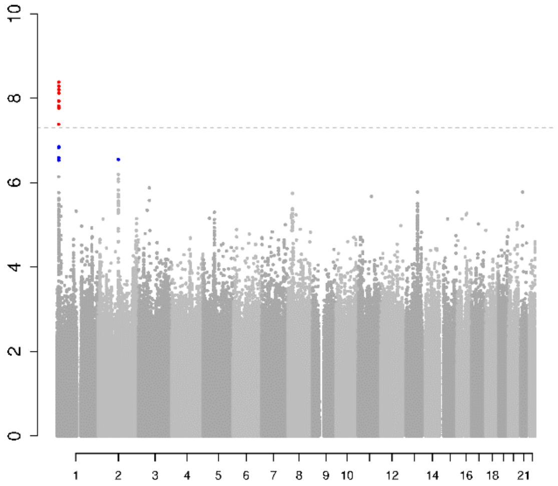

Nt-proBNP

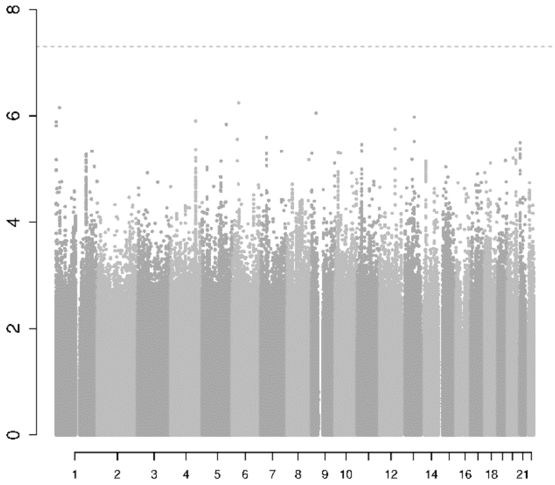

PRKACA

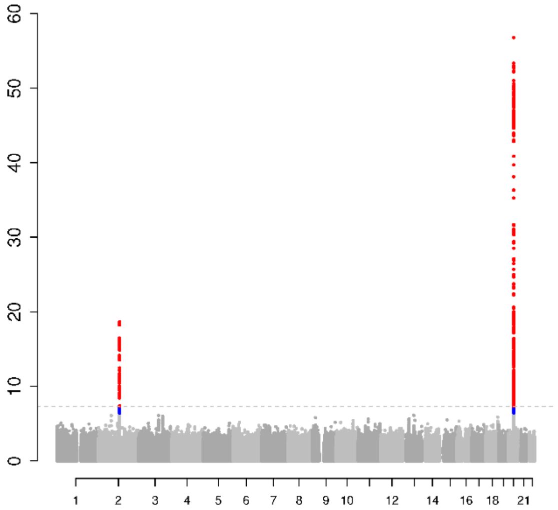

Protein C

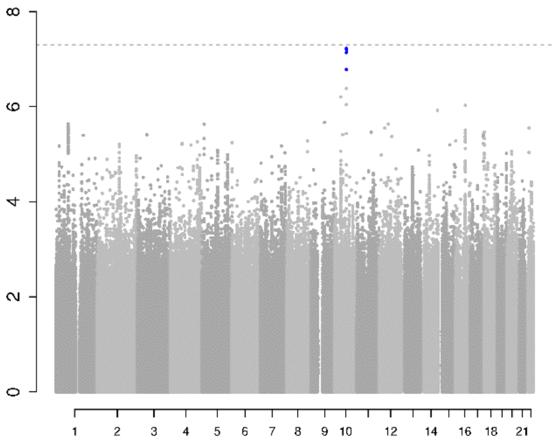

tPA

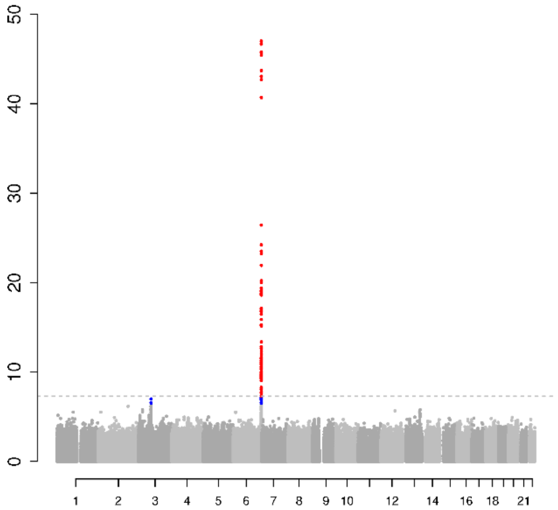

TSP2

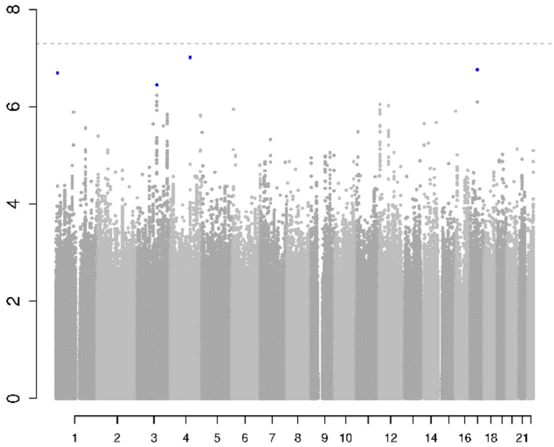

UNC5H4

**Supplementary table 1. Comparison of risk factor estimates for incident HF in the full population-based cohort and the nested case-cohort sample.**

| Covariate | Population cohort | | Nested case-cohort | |
|---|---|---|---|---|
| | **Hazard ratio** | **95% CI** | **Hazard ratio** | **95% CI** |
| Age, per year | 1.12 | 1.09-1.15 | 1.17 | 1.11-1.24 |
| Male sex | 1.83 | 1.37-2.45 | 1.96 | 1.18-3.24 |
| Systolic blood pressure, per 10 mm Hg | 1.05 | 0.96-1.15 | 1.04 | 0.86-1.27 |
| Diastolic blood pressure, per 10 mm Hg | 1.00 | 0.82-1.20 | 1.18 | 0.77-1.82 |
| Antihypertensive medications | 1.96 | 1.46-2.62 | 1.93 | 1.16-3.21 |
| History of diabetes | 2.50 | 1.68-3.70 | 2.46 | 0.96-6.30 |
| Body mass index | 1.10 | 1.07-1.13 | 1.15 | 1.08-1.21 |
| LDL cholesterol | 0.96 | 0.84-1.10 | 0.78 | 0.59-1.03 |
| HDL cholesterol | 0.75 | 0.49-1.14 | 0.82 | 0.42-1.62 |
| Current smoking | 1.80 | 1.34-2.41 | 1.88 | 1.13-3.14 |
| History of myocardial infarction | 2.26 | 1.38-3.70 | 1.04 | 0.34-3.22 |
| Nt-proBNP, standardized | 1.63 | 1.43-1.86 | 1.96 | 1.18-3.24 |

Estimates of relative risk for incident HF with individual risk factors in Prentice-weighted Cox proportional hazards regression in the nested subcohort were similar to unweighted estimates from the full population-based cohort study (Malmö Diet and Cancer Cardiovascular Cohort, n=6,103), with the exception of lack of effect of a history of myocardial infarction. The number of subjects with a history of myocardial infarction was however low in the nested case-cohort set, and overlap of confidence intervals was observed between the two cohorts.

**Supplementary table 2. Baseline characteristics of clinical heart failure cohorts.**

| | **HF outpatients** *(n=84)* | **Heart transplant recipients** *(n=30)* |
|---|---|---|
| Heart rate, (bpm) | 68 (60.75-81.25) | 77 (71-88) |
| LVEF, (%) | 30 (25-40) | 20 (10-24) |
| Medications, (n [%]) | | |
| ACE inhibitor | 66 (78.57%) | 13 (43.44%) |
| ARB | 20 (23.80%) | 14 (46.67%) |
| Betablocker | 76 (90.48%) | 28 (93.33%) |
| MRA | 48 (57.14%) | 24 (80%) |
| Loop diuretic | 65 (77.38%) | 29 (96.67%) |
| CRT, (n [%]) | 10 (11.90%) | 8 (26.67%) |
| ICD, (n [%]) | 22 (26.19%) | 24 (80%) |
| Etiology, (n [%]) | | |
| Ischemic | 28 (33.33%) | 3 (10%) |
| DCM | 33 (39.29%) | 20 (66.67%) |
| Tachy-CM | 4 (4.76%) | - |
| HCM | 3 (3.57%) | 3 (10%) |
| Myocarditis | 2 (2.38%) | 1 (3%) |
| PPCM | 2 (2.38%) | 0 |
| Other | 12 (14.29%) | 3 (10%) |
| NYHA class, (n [%]) | | |
| I | 22 (26.19%) | 1 (3%) |
| II | 37 (44.05%) | 0 |
| III | 24 (28.57%) | 27 (87%) |
| IV | - | 3 (10%) |

Median and interquartile range are presented for heart rate and LVEF. ACE, Angiotensin Converting Enzyme. ARB, Angiotensin II Receptor Blocker. CRT, cardiac resynchronization therapy. DCM, Dilated Cardiomyopathy. HCM, Hypertrophic Cardiomyopathy. HFMP, outpatient-based Heart Failure Management Program. ICD, intracardiac defibrillator. LVEF, left ventricular ejection fraction. MRA, mineralcorticoid receptor antagonists. NYHA, New York Heart Associaton. PPCM, Peripartum Cardiomyopathy. Tachy-CM, Tachycardia-induced cardiomyopathy.

**Supplementary Table 3: Association with proteins from established pathways.**

| Protein | Manifest heart failure | | Incident heart failure | |
|---|---|---|---|---|
| | *OR* | *P-value* | *HR* | *P-value* |
| *Neurohormones* | | | | |
| Angiotensinogen | 0.45 | $5x10^{-5}$ | 1.03 | 0.76 |
| Nt-ProBNP | 29.68 | $3x10^{-15}$ | 1.85 | $4x10^{-7}$ |
| Renin | 6.36 | $1x10^{-12}$ | 1.05 | 0.69 |
| *Inflammation* | | | | |
| CRP | 2.05 | $2x10^{-4}$ | 1.86 | $3x10^{-7}$ |
| TNFα | 1.29 | 0.01 | 1.13 | 0.04 |
| *Myocyte stress* | | | | |
| ST2 | 2.50 | $3x10^{-7}$ | 1.41 | 0.002 |
| Troponin T | 3.20 | $1.72.10^{-10}$ | 1.33 | 0.002 |
| *Matrix remodeling* | | | | |
| Galectin-3 | 5.15 | $8x10^{-10}$ | 1.18 | 0.11 |
| Metalloproteinases | | | | |
| MMP1 | 1.11 | 0.38 | 0.90 | 0.26 |
| MMP2 | 2.19 | $1x10^{-7}$ | 0.98 | 0.85 |
| MMP3 | 1.20 | 0.11 | 0.89 | 0.27 |
| MMP7 | 13.86 | $8x10^{-8}$ | 1.34 | 0.02 |
| MMP8 | 1.20 | 0.21 | 0.85 | 0.19 |
| MMP9 | 3.35 | $1x10^{-16}$ | 1.13 | 0.23 |
| MMP10 | 1.53 | 0.009 | 1.00 | 0.97 |
| MMP12 | 1.52 | 0.003 | 1.36 | 0.004 |
| MMP13 | 1.38 | $3x10^{-4}$ | 1.03 | 0.81 |
| MMP14 | 1.36 | 0.007 | 0.96 | 0.72 |
| MMP16 | 1.10 | 0.24 | 1.00 | 0.97 |
| MMP17 | 2.40 | 0.003 | 1.07 | 0.33 |
| MMP inhibitors | | | | |
| TIMP1 | 0.97 | 0.74 | 1.03 | 0.78 |
| TIMP2 | 2.01 | $1x10^{-4}$ | 0.86 | 0.27 |
| TIMP3 | 0.52 | $8x10^{-4}$ | 0.92 | 0.35 |

Results are presented as odds ratios (OR) or hazard ratios (HR) with p-values.

**Supplementary Table 4. Association with manifest heart failure for proteins identified for heart failure development.**

| Protein | Nested population cohort (n=583) | Incident HF (n=185) | Manifest HF outpatients (n=84) | P-value | Protein class | Subcellular location | Tissue enrichment |
|---|---|---|---|---|---|---|---|
| *Vascular messengers* | | | | | | | |
| Nt-proBNP | 4,089 (2,351-5,981) | 5,615 (3,281-11,537) | 38,630 (16,352-72,782) | <0.001 | Secreted | | Tissue enriched |
| *Matrix remodelling* | | | | | | | |
| TSP2 | 12,930 (10,462-15,771) | 14,592 (11,800-18,667) | 20,051 (13,371-32,780) | <0.001 | Secreted | Nuclear speckles, plasma membrane, cytosol | Tissue enhanced |
| *Immune system* | | | | | | | |
| CXCL13 | 2,011 (1,672-2,479) | 2,302 (1,967-2,996) | 2,599 (2,078-3,438) | <0.001 | Secreted | | Group enriched |
| CRP | 43,186 (25,712-73,110) | 70,995 (40,625-98,303) | 82,280 (48,078-118,930) | <0.001 | Secreted | | Group enriched |
| IL-1R antagonist protein | 3,966 (3,441-4,811) | 4,506 (3,833-5,580) | 4,982 (3,739-6,660) | <0.001 | Secreted | | Tissue enhanced |
| IL-18 receptor 1 | 9,274 (7,976-10,867) | 10,225 (8,658-12,315) | 12,062 (10,254-13,408) | <0.001 | Secreted, Membrane | Mitochondria | Mixed |
| *Complement system* | | | | | | | |
| C5a | 11,621 (9,491-14,956) | 14,075 (11,003-18,282) | 18,478 (12,977-23,682) | <0.001 | Secreted | | Tissue enriched |
| C9 | 68,658 (61,101-76,994) | 74,440 (66,080-84,930) | 85,160 (68,345-95,255) | <0.001 | Secreted | Plasma membrane, cytosol | Tissue enriched |
| *Coagulation system* | | | | | | | |
| Protein C | 1,685 (1,561-1,854) | 1,619 (1,486-1,811) | 1,451 (900-1,640) | <0.001 | Secreted | | Tissue enriched |

| | | | | | | | |
|---|---|---|---|---|---|---|---|
| tPA | 320 (259-400) | 379 (297-488) | 499 (370-639) | <0.001 | Secreted | Actin filaments | Tissue enriched |
| *Intracellular or membrane-bound* | | | | | | | |
| Gelsolin | 954 (866-1,035) | 890 (790-984) | 806 (722-913) | <0.001 | Secreted | Actin filaments | Expressed in all |
| Carbonic anhydrase 13 | 9,336 (6,732-13,319) | 7,277 (5,129-10,709) | 2,126 (1,150-3,468) | <0.001 | Intracellular | Nucleus, nucleoli, vesicles | Mixed |
| Contactin-1 | 282 (254-310) | 261 (236-291) | 240 (213-273) | <0.001 | Secreted | | Tissue enhanced |
| DUSP3 | 4,956 (3,735-6,350) | 3,930 (3,084-5,600) | 1,227 (811-1,861) | <0.001 | Intracellular | Nucleoplasm, cytosol | Expressed in all |
| PRKACA | 9,280 (7,099-12,312) | 8,222 (5,863-10,889) | 1,846 (1,354-3,100) | <0.001 | Intracellular | Cytosol | Expressed in all |
| Netrin receptor UNC5D | 4,816 (4,002-5,762) | 4,320 (3,615-5,050) | 5,274 (4,042-6,651) | 0.08 | Membrane | | Tissue enhanced |

Values in relative fluorescent units from the general population cohort and cases with manifest heart failure presented as median and interquartile range. P-values for difference between subjects from the general population and manifest heart failure were computed using linear regression of log-transformed concentrations adjusted for age and sex. Information on protein class (intracellular, membrane-bound, secreted, unclassified) and subcellular location (cytosol, nucleus, vesicles, Golgi apparatus, etc) was obtained from the Human Protein Atlas (HPA, https://www.proteinatlas.org, accessed sept 9, 2018). Information on tissue enrichment was also obtained from the HPA, based on RNA-sequencing of 64 cell lines and 37 tissues. Tissue expression was classified by the HPA into six categories: tissue enriched (expression in one tissue at least five-fold higher than all others), group enriched (five-fold higher in 2-7 tissues compared to all other), tissue enhanced (five-fold higher in one or more tissues compared to the mean of all tissues), expressed in all (≥1 transcript per million in all tissues), not detected (<1 transcript per million in all tissues), and mixed (detected in at least one tissue and not in the other categories).

**Supplementary table 5. Correlation of proteins associated with heart failure development.**

| | C5a | Gelsolin | CXCL13 | TSP2 | Protein C | CRP | Nt-proBNP | CA13 | Contactin-1 | C9 | DUSP3 | tPA | IL-1RA | IL-18R1 | PRKACA | Netrin receptor UNC5D |
|---|---|---|---|---|---|---|---|---|---|---|---|---|---|---|---|---|
| C5a | 1.00 | | | | | | | | | | | | | | | |
| Gelsolin | **-0.43** | 1.00 | | | | | | | | | | | | | | |
| CXCL13 | 0.06 | -0.15 | 1.00 | | | | | | | | | | | | | |
| TSP2 | 0.12 | -0.15 | 0.29 | 1.00 | | | | | | | | | | | | |
| Protein C | 0.06 | -0.04 | -0.20 | **-0.31** | 1.00 | | | | | | | | | | | |
| CRP | **0.58** | **-0.41** | 0.17 | 0.20 | -0.09 | 1.00 | | | | | | | | | | |
| Nt-proBNP | -0.12 | 0.14 | 0.21 | **0.30** | -0.20 | 0.01 | 1.00 | | | | | | | | | |
| CA13 | -0.20 | 0.24 | -0.14 | -0.24 | 0.21 | -0.22 | -0.08 | 1.00 | | | | | | | | |
| Contactin-1 | **-0.40** | **0.38** | -0.10 | -0.03 | 0.006 | **-0.36** | 0.10 | 0.18 | 1.00 | | | | | | | |
| C9 | **0.33** | -0.19 | 0.14 | 0.05 | -0.16 | **0.34** | 0.14 | -0.06 | -0.23 | 1.00 | | | | | | |
| DUSP3 | -0.28 | 0.21 | -0.18 | -0.27 | 0.19 | -0.25 | -0.10 | **0.82** | 0.12 | -0.10 | 1.00 | | | | | |
| tPA | 0.03 | -0.15 | 0.11 | 0.18 | -0.10 | 0.20 | 0.10 | -0.24 | -0.01 | -0.19 | -0.22 | 1.00 | | | | |
| IL-1RA | 0.20 | -0.17 | 0.12 | 0.28 | -0.17 | 0.29 | 0.03 | -0.13 | -0.19 | 0.008 | -0.14 | 0.14 | 1.00 | | | |
| IL-18 receptor 1 | 0.20 | -0.19 | 0.24 | **0.48** | -0.23 | 0.22 | 0.14 | **-0.32** | -0.09 | 0.0007 | **-0.34** | 0.19 | 0.29 | 1.00 | | |
| PRKACA | -0.27 | **0.31** | -0.21 | -0.20 | 0.12 | -0.22 | -0.07 | **0.75** | 0.18 | -0.13 | **0.74** | -0.17 | -0.10 | -0.28 | 1.00 | |
| Netrin receptor UNC5D | -0.19 | 0.28 | 0.02 | 0.10 | -0.10 | -0.23 | 0.26 | -0.007 | 0.18 | 0.02 | -0.07 | -0.14 | 0.003 | 0.12 | 0.02 | 1.00 |

Pairwise correlation of log-transformed proteins associated with heart failure, expressed as Pearson’s correlation coefficients. Coefficients >0.3 have been indicated in bold. All correlation coefficients >0.07 were had p<0.05. CA13, Carbonic anhydrase 13. IL-1RA, IL-1R antagonist protein.

**Supplementary Table 6: Gene set enrichment analysis for manfiest heart failure.**

| Gene Set | Genes, total (K) | Genes, overlapping (k) | k/K | FDR q-value | Description |
|---|---|---|---|---|---|
| HALLMARK_PI3K_AKT_MTOR_SIGNALING | 105 | 26 | 0.2476 | $6x10^{-28}$ | Genes up-regulated by activation of the PI3K/AKT/mTOR pathway. |
| HALLMARK_COMPLEMENT | 200 | 29 | 0.1450 | $1x10^{-24}$ | Genes encoding components of the complement system, which is part of the innate immune system. |
| HALLMARK_EPITHELIAL_MESENCHYMAL_TRANSITION | 200 | 29 | 0.1450 | $1x10^{-24}$ | Genes defining epithelial-mesenchymal transition, as in wound healing, fibrosis and metastasis. |
| HALLMARK_COAGULATION | 138 | 23 | 0.1667 | $5x10^{-21}$ | Genes encoding components of blood coagulation system; also up-regulated in platelets. |
| HALLMARK_IL6_JAK_STAT3_SIGNALING | 87 | 19 | 0.2184 | $7x10^{-20}$ | Genes up-regulated by IL6 [GeneID=3569] via STAT3 [GeneID=6774], e.g., during acute phase response. |
| HALLMARK_ALLOGRAFT_REJECTION | 200 | 25 | 0.1250 | $8x10^{-20}$ | Genes up-regulated during transplant rejection. |
| HALLMARK_INFLAMMATORY_RESPONSE | 200 | 20 | 0.1000 | $4x10^{-14}$ | Genes defining inflammatory response. |
| HALLMARK_ANGIOGENESIS | 36 | 11 | 0.3056 | $1x10^{-13}$ | Genes up-regulated during formation of blood vessels (angiogenesis). |
| HALLMARK_HYPOXIA | 200 | 19 | 0.0950 | $3x10^{-13}$ | Genes up-regulated in response to low oxygen levels (hypoxia). |
| HALLMARK_TNFA_SIGNALING_VIA_NFKB | 200 | 19 | 0.0950 | $3x10^{-13}$ | Genes regulated by NF-kB in response to TNF [GeneID=7124]. |
| HALLMARK_APICAL_JUNCTION | 200 | 17 | 0.0850 | $3x10^{-11}$ | Genes encoding components of apical junction complex. |
| HALLMARK_GLYCOLYSIS | 200 | 17 | 0.0850 | $3x10^{-11}$ | Genes encoding proteins involved in glycolysis and gluconeogenesis. |
| HALLMARK_MYC_TARGETS_V1 | 200 | 17 | 0.0850 | $3x10^{-11}$ | A subgroup of genes regulated by MYC - version 1 (v1). |
| HALLMARK_XENOBIOTIC_METABOLISM | 200 | 17 | 0.0850 | $3x10^{-11}$ | Genes encoding proteins involved in processing of drugs and other xenobiotics. |
| HALLMARK_INTERFERON_GAMMA_RESPONSE | 200 | 16 | 0.0800 | $3x10^{-10}$ | Genes up-regulated in response to IFNG [GeneID=3458]. |
| HALLMARK_KRAS_SIGNALING_UP | 200 | 16 | 0.0800 | $3x10^{-10}$ | Genes up-regulated by KRAS activation. |
| HALLMARK_MTORC1_SIGNALING | 200 | 16 | 0.0800 | $3x10^{-10}$ | Genes up-regulated through activation of mTORC1 complex. |
| HALLMARK_APICAL_SURFACE | 44 | 9 | 0.2045 | $7x10^{-10}$ | Genes encoding proteins over-represented on the apical surface of epithelial cells, e.g., important for cell polarity (apical area). |
| HALLMARK_FATTY_ACID_METABOLISM | 158 | 14 | 0.0886 | $9x10^{-10}$ | Genes encoding proteins involved in metabolism of fatty acids. |

| | | | | | |
|---|---|---|---|---|---|
| HALLMARK_APOPTOSIS | 161 | 14 | 0.0870 | $1x10^{-9}$ | Genes mediating programmed cell death (apoptosis) by activation of caspases. |
| HALLMARK_IL2_STAT5_SIGNALING | 200 | 15 | 0.0750 | $2x10^{-9}$ | Genes up-regulated by STAT5 in response to IL2 stimulation. |
| HALLMARK_UV_RESPONSE_UP | 158 | 13 | 0.0823 | $8x10^{-9}$ | Genes up-regulated in response to ultraviolet (UV) radiation. |
| HALLMARK_ADIPOGENESIS | 200 | 12 | 0.0600 | $1x10^{-6}$ | Genes up-regulated during adipocyte differentiation (adipogenesis). |
| HALLMARK_MYOGENESIS | 200 | 12 | 0.0600 | $1x10^{-6}$ | Genes involved in development of skeletal muscle (myogenesis). |
| HALLMARK_P53_PATHWAY | 200 | 11 | 0.0550 | $7x10^{-6}$ | Genes involved in p53 pathways and networks. |
| HALLMARK_UV_RESPONSE_DN | 144 | 9 | 0.0625 | $2x10^{-5}$ | Genes down-regulated in response to ultraviolet (UV) radiation. |
| HALLMARK_ESTROGEN_RESPONSE_LATE | 200 | 10 | 0.0500 | $4x10^{-5}$ | Genes defining late response to estrogen. |
| HALLMARK_G2M_CHECKPOINT | 200 | 9 | 0.0450 | $2x10^{-4}$ | Genes involved in the G2/M checkpoint, as in progression through the cell division cycle. |
| HALLMARK_ANDROGEN_RESPONSE | 101 | 6 | 0.0594 | $7x10^{-4}$ | Genes defining response to androgens. |
| HALLMARK_ESTROGEN_RESPONSE_EARLY | 200 | 8 | 0.0400 | 0.001 | Genes defining early response to estrogen. |
| HALLMARK_TGF_BETA_SIGNALING | 54 | 4 | 0.0741 | 0.003 | Genes up-regulated in response to TGFB1 [GeneID=7040]. |
| HALLMARK_PROTEIN_SECRETION | 96 | 5 | 0.0521 | 0.003 | Genes involved in protein secretion pathway. |
| HALLMARK_INTERFERON_ALPHA_RESPONSE | 97 | 5 | 0.0515 | 0.003 | Genes up-regulated in response to alpha interferon proteins. |
| HALLMARK_HEME_METABOLISM | 200 | 7 | 0.0350 | 0.004 | Genes involved in metabolism of heme (a cofactor consisting of iron and porphyrin) and erythroblast differentiation. |
| HALLMARK_NOTCH_SIGNALING | 32 | 3 | 0.0938 | 0.005 | Genes up-regulated by activation of Notch signaling. |
| HALLMARK_HEDGEHOG_SIGNALING | 36 | 3 | 0.0833 | 0.006 | Genes up-regulated by activation of hedgehog signaling. |
| HALLMARK_PANCREAS_BETA_CELLS | 40 | 3 | 0.0750 | 0.008 | Genes specifically up-regulated in pancreatic beta cells. |
| HALLMARK_REACTIVE_OXIGEN_SPECIES_PATHWAY | 49 | 3 | 0.0612 | 0.01 | Genes up-regulated by reactive oxigen species (ROS). |
| HALLMARK_KRAS_SIGNALING_DN | 200 | 6 | 0.0300 | 0.01 | Genes down-regulated by KRAS activation. |
| HALLMARK_MYC_TARGETS_V2 | 58 | 3 | 0.0517 | 0.02 | A subgroup of genes regulated by MYC - version 2 (v2). |
| HALLMARK_CHOLESTEROL_HOMEOSTASIS | 74 | 3 | 0.0405 | 0.04 | Genes involved in cholesterol homeostasis. |
| HALLMARK_E2F_TARGETS | 200 | 5 | 0.0250 | 0.05 | Genes encoding cell cycle related targets of E2F transcription factors. |
| HALLMARK_MITOTIC_SPINDLE | 200 | 5 | 0.0250 | 0.05 | Genes important for mitotic spindle assembly. |

Pathways enriched (false discovery rate [FDR] <0.05) among proteins associated with manifest heart failure in gene-set enrichment analysis (GSEA) of hallmark pathways from the Molecular Signatures Database.

**Supplementary Table 7. Evidence of cardiac origin.**

| Protein | Coronary sinus | Femoral vein | Ratio | Cardiac expression |
|---|---|---|---|---|
| *Vascular messengers* | | | | |
| Nt-proBNP | - | - | - | G, Hg, N/A, M |
| *Matrix remodelling* | | | | |
| TSP2 | 19,903 (10,844-29,252) | 18,922 (10,248-30,996) | 1.05 | G, Hg, Hi, M |
| *Immune system* | | | | |
| CXCL13 | 6,486 (2,973-9,651) | 7,348 (2,849-9,515) | 0.88 | -, -, -, - |
| CRP | 36,630 (34,278-40,330) | 37,878 (36,402-39,364) | 0.97 | -, -, -, M |
| IL-1R antagonist protein | - | - | - | -, -, -, M |
| IL-18 receptor 1 | 11,827 (8,650-13,962) | 11,552 (8,422-12,671) | 1.02 | G, Hg, Hi, M |
| *Complement system* | | | | |
| C5a | 14,576 (10,762-15,988) | 12,855 (10,175-18,259) | 1.13 | N/A, M |
| C9 | 73,155 (63,848-102,885) | 82,635 (64,692-100,912) | 0.89 | -, -, -, M |
| *Coagulation system* | | | | |
| Protein C | 1,734 (1,646-2,083) | 1,747 (1,650-2,029) | 0.99 | -, -, -, M |
| tPA | 843 (646-920) | 792 (682-905) | 1.06 | -, -, -, M |
| *Intracellular or membrane-bound* | | | | |
| Gelsolin | 795 (777-882) | 768 (747-900) | 1.04 | G, Hg, -, M |
| Carbonic anhydrase 13 | 881 (689-1,055) | 939 (868-1,030) | 0.94 | G, Hg, Hi, M |
| Contactin-1 | 311 (277-352) | 335 (278-258) | 0.93 | -, -, -, M |
| DUSP3 | 560 (480-900) | 622 (555-768) | 0.90 | G, Hg, N/A, M |
| PRKACA | 1,346 (1,241-1,463) | 1,530 (1,154-1,796) | 0.88 | G, Hg, Hi, M |
| Netrin receptor UNC5D | 5,068 (4,667-7,901) | 4,616 (4,072-7,944) | 1.10 | -, -, N/A, - |

Samples obtained from patients with heart failure due to hypertrophic cardiomyopathy with outflow obstruction before underoing alcohol septal ablation. Abundance expressed in relative fluorescent units from coronary sinus and femoral vein plasma presented as median (25th-75th percentile). Paired measures from the two sampling sites were compared with the Wilcoxon signed-rank test. All proteins except Nt-proBNP and IL-1RA were available on the older version of the aptamer-based proteomic assay used in this cohort. For cardiac expression, 'G' indicates cardiac gene expression in GTEx, 'Hg' cardiac gene expression in the Human Protein Atlas, 'Hi' cardiac protein expression according to immunohistochemistry in the Human Protein Atlas, and 'M' cardiac protein expression according to mass spectrometry. N/A indicates that the protein was not available in the immunohistochemistry data, and for C5a that is was also not available in the gene expression datasets due to the post-translational processing necessary for generation of this protein.

**Supplementary Table 8: Results from genome-wide association analyses.**

| Protein | Location | Locus | Position | SNP | Beta | SE | P-value | cA1 | A2 | CAF | |
|---|---|---|---|---|---|---|---|---|---|---|---|
| Nt-proBNP | cis | 1p36 | 11,915,467 | rs198379 | 0.22 | 0.03 | $1x10^{-13}$ | T | C | 0.60 | * |
| TSP2 | Cis | 6q27 | 169,624,770 | rs74507247 | 0.83 | 0.06 | $2x10^{-47}$ | A | G | 0.11 | |
| CXCL13 | - | - | | - | - | - | - | - | - | - | |
| CRP | cis | 1q23 | 159,693,605 | rs2211320 | -0.23 | 0.04 | $6x10^{-10}$ | A | G | 0.34 | |
| IL-1RAP | cis | 2q13 | 113,834,434 | rs12468224 | 0.32 | 0.04 | $9x10^{-17}$ | G | C | 0.44 | |
| IL-18R1 | cis | 2q12 | 102,991,181 | rs6731154 | 0.87 | 0.04 | $9x10^{-135}$ | C | T | 0.27 | |
| C5a | cis | 9q33 | 123,778,148 | rs10116271 | 0.16 | 0.03 | $3x10^{-10}$ | T | C | 0.51 | * |
| | trans | 1q31 | 196,664,793 | rs485632 | 0.25 | 0.04 | $2x10^{-10}$ | T | G | 0.40 | |
| C9 | cis | 5p13 | 39,327,888 | rs62358361 | -1.62 | 0.26 | $4x10^{-10}$ | T | G | 0.01 | |
| Protein C | cis | 2q14 | 128,165,478 | rs61185143 | -0.35 | 0.04 | $2x10^{-19}$ | T | C | 0.43 | |
| | trans | 20q11 | 33,784,021 | rs11906148 | 0.90 | 0.06 | $2x10^{-57}$ | A | C | 0.10 | |
| tPA | cis | 8p11 | 42,008,698 | rs182065212 | 0.57 | 0.11 | 1x10-7 | C | G | 0.98 | * |
| Gelsolin | cis | 9q33 | 124,103,182 | rs41273436 | -0.80 | 0.13 | 2x10-9 | T | C | 0.99 | * |
| Carbonic anhydrase 13 | cis | 8q21 | 86,139,240 | rs6987314 | -0.66 | 0.07 | $2x10^{-19}$ | A | G | 0.08 | |
| Contactin-1 | cis | 12q12 | 41,186,595 | rs11178396 | 0.25 | 0.04 | $2x10^{-11}$ | C | A | 0.34 | |
| | trans | 16p13 | 420,907 | rs540 | 0.31 | 0.04 | $1x10^{-17}$ | T | C | 0.42 | |
| DUSP3 | - | - | - | - | - | - | - | - | - | - | |
| PRKACA | - | - | - | - | - | - | - | - | - | - | |
| UNC5D | cis | 8p12 | 35,035,535 | rs6468309 | -0.15 | 0.03 | 1x10-6 | A | G | 0.54 | * |

Results from genome-wide association analyses in the Malmö Diet and Cancer Study of 16 proteins associated with incident heart failure. Results from the index SNP, based on lowest p-value, at each locus. Beta coefficients with standard errors (SE) and p-value from general linear models adjusted for age and sex, as implemented in the R package GWAF. Coded allele (cA1), non-coded allele (A2) and coded allele frequency (CAF) are also presented. Positions refer to NCBI 37. * Asterisk indicates loci that were discovered in a second stages focused on discovery of cis-regulatory variants for the 7 proteins for which such variants were not discovered in the Malmö Diet and Cancer study. This analysis was conducted in the combination of Malmö Diet and Cancer results with those from a previously published analysis of the Framingham Heart Study (Benson M et al, Circulation 2018;137:1158-72). The number of SNPs tested in this analysis was smaller and a more permissive significance treshold was used.
