# Supplementary material for: Profiling of the plasma proteome across different stages of human heart failure
Source: Nat Commun. 2019 Dec 20;10:5830. doi: 10.1038/s41467-019-13306-y (PMC6925199; doi:10.1038/s41467-019-13306-y)
Supplement: Supplementary file 3 — Description of Additional Supplementary Files [file 41467_2019_13306_MOESM3_ESM.docx]

**Description of Supplementary Files**

**File Name: Supplementary Data 1**

**Description:** Proteins assayed by the aptamer-based proteomics assay.

**File Name: Supplementary Data 2**

**Description:** Proteins associated with heart failure development.

**File Name: Supplementary Data 3**

**Description:** Proteins associated with manifest heart failure.

**File Name: Supplementary Data 4**

**Description:** Proteins associated with manifest heart failure and transplantation dynamics.

**File Name: Supplementary Data 5**

**Description:** Proteins before and after heart transplantation.
