# Supplementary material for: Profiling of the plasma proteome across different stages of human heart failure
Source: Nat Commun. 2019 Dec 20;10:5830. doi: 10.1038/s41467-019-13306-y (PMC6925199; doi:10.1038/s41467-019-13306-y)
Supplement: Supplementary file 9 — Reporting Summary [file 41467_2019_13306_MOESM9_ESM.pdf]

nature research

Corresponding author(s): J. Gustav Smith

Reporting Summary

Nature Research wishes to improve the reproducibility of the work that we publish. This form provides structure for consistency and transparency in reporting. For further information on Nature Research policies, see Authors & Referees and the Editorial Policy Checklist.

Statistical parameters

When statistical analyses are reported, confirm that the following items are present in the relevant location (e.g. figure legend, table legend, main text, or Methods section).

n/a	Confirmed	
☐	☒	The exact sample size (*n*) for each experimental group/condition, given as a discrete number and unit of measurement
☐	☒	An indication of whether measurements were taken from distinct samples or whether the same sample was measured repeatedly
☐	☒	The statistical test(s) used AND whether they are one- or two-sided<br>*Only common tests should be described solely by name; describe more complex techniques in the Methods section.*
☐	☒	A description of all covariates tested
☐	☒	A description of any assumptions or corrections, such as tests of normality and adjustment for multiple comparisons
☐	☒	A full description of the statistics including central tendency (e.g. means) or other basic estimates (e.g. regression coefficient) AND variation (e.g. standard deviation) or associated estimates of uncertainty (e.g. confidence intervals)
☐	☒	For null hypothesis testing, the test statistic (e.g. *F*, *t*, *r*) with confidence intervals, effect sizes, degrees of freedom and *P* value noted<br>*Give P values as exact values whenever suitable.*
☒	☐	For Bayesian analysis, information on the choice of priors and Markov chain Monte Carlo settings
☒	☐	For hierarchical and complex designs, identification of the appropriate level for tests and full reporting of outcomes
☐	☒	Estimates of effect sizes (e.g. Cohen's *d*, Pearson's *r*), indicating how they were calculated
☐	☒	Clearly defined error bars<br>*State explicitly what error bars represent (e.g. SD, SE, CI)*

Our web collection on statistics for biologists may be useful.

Software and code

Policy information about availability of computer code

Data collection	n/a
Data analysis	Statistical analyses were conducted using STATA version 12 (StataCorp, College Station, Texas, USA), the GSEA software as implemented online (http://software.broadinstitute.org/gsea/msigdb/index.jsp), and the R package GWAF v2.2 (https://cran.r-project.org/web/packages/GWAF/index.html).

For manuscripts utilizing custom algorithms or software that are central to the research but not yet described in published literature, software must be made available to editors/reviewers upon request. We strongly encourage code deposition in a community repository (e.g. GitHub). See the Nature Research guidelines for submitting code & software for further information.

Data

Policy information about availability of data

All manuscripts must include a data availability statement. This statement should provide the following information, where applicable:

- Accession codes, unique identifiers, or web links for publicly available datasets
- A list of figures that have associated raw data
- A description of any restrictions on data availability

All the relevant data supporting the findings of this study are available within this article, in the supplementary materials and source data, or can be obtained from

referenced respositories.

Field-specific reporting

Please select the best fit for your research. If you are not sure, read the appropriate sections before making your selection.

- [x] Life sciences
- [ ] Behavioural & social sciences
- [ ] Ecological, evolutionary & environmental sciences

For a reference copy of the document with all sections, see nature.com/authors/policies/ReportingSummary-flat.pdf

Life sciences study design

All studies must disclose on these points even when the disclosure is negative.

Sample size	Sample based on population-based and clinical cohorts from biobanks. All eligible participants with phenotypic data available included in the present analyses.
Data exclusions	No exclusions.
Replication	Strict significance thresholds based on Bonferroni-correction were applied. Proteins meeting this threshold were followed up in independent cohorts with manifest heart failure and heart failure patients undergoing transplantation. Results for manifest heart failure were also compared to another smaller study.
Randomization	Population-based cohorts were used. Potential confounding factors were evaluated in a series of tests, including analysis after heart transplantation, blood samples obtained from the cardiac coronary sinus, adjustment for potential confounders in regression models, exclusion in sensitivity analyses of individuals on certain medications such as warfarin that could influence protein profiles, genome-wide association studies to evaluate specificity of reagents.
Blinding	At the timepoint of the collection of the samples used here into biobanks, study personnel were unaware of the current study and were completely blinded to outcomes and protein concentrations.

Reporting for specific materials, systems and methods

Materials & experimental systems

n/a	Involved in the study	
☐	☒	Unique biological materials
☒	☐	Antibodies
☒	☐	Eukaryotic cell lines
☒	☐	Palaeontology
☒	☐	Animals and other organisms
☐	☒	Human research participants

Methods

n/a	Involved in the study	
☒	☐	ChIP-seq
☒	☐	Flow cytometry
☒	☐	MRI-based neuroimaging

Unique biological materials

Policy information about availability of materials

Obtaining unique materials	Blood samples from all cohorts used can be obtained by reasonable written requests to the corresponding author, as long as there are remaining sample volumes.

Human research participants

Policy information about studies involving human research participants

Population characteristics	Plasma samples were studied from a total of 583 population-based subjects and 185 subjects with incident HF, all from the Malmö Diet and Cancer study (average age 57 and 61 years; 59% and 43% female). In addition, plasma samples from 84 patients with advanced heart failure, 30 patients undergoing heart transplantation, 6 subjects undergoing septal ablation, and heart samples from 2 subjects with advanced heart failure undergoing heart transplantation were used. Detailed characteristics of all cohorts are provided in Table 1 and Supplementary Table 2.
Recruitment	The Malmö Diet and Cancer study invited all men (born between 1923–1945) and women (born between 1923–1950) from the city of Malmö in southern Sweden to attend baseline exams in 1991-1996. All consecutive patients with advanced heart failure attending the outpatient clinic at our hospital in 2013-14 and all undergoing heart transplantation between 2012-2016 were invited to participate in the clinical biobank. Detailed descriptions of the recruitment have been published and are referenced in the Methods section.
